# Glutamine Codon Usage and polyQ Evolution in Primates Depend on the Q Stretch Length

**DOI:** 10.1093/gbe/evy046

**Published:** 2018-03-01

**Authors:** Pablo Mier, Miguel A Andrade-Navarro

**Affiliations:** 1 Faculty of Biology, Johannes Gutenberg University Mainz, Germany; 2 Institute of Molecular Biology, Mainz, Germany

**Keywords:** homorepeat, glutamine stretch, codon usage, polyQ-associated diseases

## Abstract

Amino acid usage in a proteome depends mostly on its taxonomy, as it does the codon usage in transcriptomes. Here, we explore the level of variation in the codon usage of a specific amino acid, glutamine, in relation to the number of consecutive glutamine residues. We show that CAG triplets are consistently more abundant in short glutamine homorepeats (polyQ, four to eight residues) than in shorter glutamine stretches (one to three residues), leading to the evolutionary growth of the repeat region in a CAG-dependent manner. The length of orthologous polyQ regions is mostly stable in primates, particularly the short ones. Interestingly, given a short polyQ the CAG usage is higher in unstable-in-length orthologous polyQ regions. This indicates that CAG triplets produce the necessary instability for a glutamine stretch to grow. Proteins related to polyQ-associated diseases behave in a more extreme way, with longer glutamine stretches in human and evolutionarily closer nonhuman primates, and an overall higher CAG usage. In the light of our results, we suggest an evolutionary model to explain the glutamine codon usage in polyQ regions.

## Introduction

Homorepeats are defined as runs of the same amino acid in a protein sequence. Given the repeated amino acid X, its homorepeat is known as polyX, X-AAR (amino acid repeat), or X-homopeptide (Zhou et al. 2011). The prevalence and functions of a polyX vary in proteomes depending on known (natural selection, taxonomy, length, GC content) (Faux et al. 2005; Mularoni et al. 2010; Zhou et al. 2011; Mier et al. 2017) and unknown factors (e.g., the case of poly-asparagines in the amoeba *Dictyostelium discoideum* and the protozoan *Plasmodium falciparum*) (Eichinger et al. 2005; Muralidharan and Goldberg 2013).

From a purely anthropocentric point of view, the most interesting homorepeats are the poly-glutamines (polyQ). Besides being one of the most prevalent homorepeats in eukaryotes (Faux et al. 2005; Mier et al. 2017), abnormal expansion of glutamine tracts (via CAG trinucleotide repeats) are associated with at least nine inherited neurodegenerative diseases (Fan et al. 2014; Den Dunnen 2017). None of these diseases are neither curable nor effectively treatable so far, despite the many attempts to fathom the role of the extended polyQ in the progression of the disorder and the development of potential therapeutic approaches (Hughes and Olson 2001; Robertson and Bottomley 2010; Margulis et al. 2013; Fan et al. 2014; Takeuchi et al. 2014; Takeuchi and Nagai 2017).

Intrinsically, the presence of CAG and other CNG repeats affects mRNA stability and structure (Broda et al. 2005), and their abnormal expansion in disease can influence splicing (Neueder et al. 2017). On the other hand, at the protein level the length of a polyQ region correlates with its propensity to aggregate (Barton et al. 2007), and is a critical determinant of age-of-disease onset (Nagai et al. 2000). These facts underline the importance of a better comprehension of the evolutionary perspective of the growth of glutamine tracts and its codon usage.

Although polyQ is a common accepted term for stretches of consecutive glutamine residues, thresholds of a minimum of four out of five (UniProt, http://www.uniprot.org/help/compbias; last accessed February 26, 2018), four out of six (Mier and Andrade-Navarro 2017), five (Albà and Guigó 2004; Jorda and Kajava 2010; Chavali et al. 2017), six (Lobanov and Galzitskaya 2012), and eight out of ten (Schaefer et al. 2012; Mier and Andrade-Navarro 2016) glutamine residues have been used so far to refer to polyQ regions. Although it has been demonstrated that for human and metazoan proteomes a stretch of five consecutive glutamines is not a random feature and thus can be considered a polyQ (Lobanov et al. 2016), we showed in a previous research (Totzeck et al. 2017) that a protein sequence with a minimum of four glutamines in a window of six amino acids already possesses characteristic features of a polyQ region. To study the maximum number of glutamine stretches but also to account for the functional and structural implications of a polyQ, we consider that: a glutamine stretch may be considered for *Q* ≥ 1, a polyQ region may be considered for *Q* ≥ 4, a short polyQ has a defined length of 4 ≤ *Q* ≤ 8, and a long polyQ is *Q* ≥ 9 residues long. We note that these thresholds are likely specific to polyQ and might not apply to other homorepeats, as the properties of homorepeats are highly influenced by the repeated residue type (Bernacki and Murphy 2011; Lu and Murphy 2015).

Glutamine is coded by synonymous codons CAA and CAG. Codon usage biases are organism- or taxa-specific and are affected by natural selection (Lynn et al. 2002; Athey et al. 2017). Codon optimality derived from these biases is a major determinant of mRNA stability (Presnyak et al. 2015) and controls mRNA translation (Saikia et al. 2016). In primates, CAG is roughly three times more frequent than CAA (35.28 and 13.66 per 1,000 codons, respectively) (Athey et al. 2017), driving the glutamines to be coded by a 72.09% CAG (71.85% CAG in human). These numbers do not consider any additional feature of the coded glutamine, like if it is influenced by the presence of adjacent glutamine residues.

In this work, we characterize the length-dependent codon usage of glutamine in glutamine stretches from complete proteomes of diverse taxonomic lineages. Focusing on orthologous Q stretches from primates, their length differences and the codon usage of stable- versus unstable-in-length stretches are assessed. We also show how glutamine stretches in proteins related to polyQ-associated diseases deviate from the expected proteome-wide codon usage, and propose an evolutionary model to explain the glutamine codon usage in polyQ regions.

## Materials and Methods

### Data Retrieval

We downloaded all coding and peptide sequences from protein coding genes from the human data set GRCh38.p10 using Ensembl/Biomart version 90 (Yates et al. 2016). Similar information was retrieved for all nonhuman primates for which Ensembl provides information about orthology relationships with human sequences: *Pan troglodytes* (ptr, CHIMP2.1.4), *Gorilla gorilla gorilla* (ggo, gorGor3.1), *Pongo abelii* (pab, PPYG2), *Nomascus leucogenys* (nle, Nleu1.0), *Macaca mulatta* (mmul, Mmul8.0.1), *Chlorocebus sabaeus* (csa, ChlSab1.1), *Papio anubis* (pan, PanAnu2.0), *Callithrix jacchus* (cja, C_jacchus3.2.1), *Carlito syrichta* (csy, tarSyr1), *Otolemur garnettii* (oga, OtoGar3), and *Microcebus murinus* (mmur, Mmur2.0).

The downloaded data were complemented with coding and peptide sequences from protein coding genes of model organisms from different taxonomic groups available in Ensembl/Biomart version 90: *Mus musculus* (mmu, GRCm38.p5), *Rattus norvegicus* (rno, Rnor6.0), *Sus scrofa* (ssc, Sscrofa11.1), *Monodelphis domestica* (mdo, monDom5), *Gallus gallus* (gga, Gallus_gallus-5.0), *Taeniopygia guttata* (tgu, taeGut3.2.4), *Xenopus tropicalis* (xtr, JGI 4.2), *Latimeria chalumnae* (lch, LatCha1), *Danio rerio* (dre, GRCz10), *Takifugu rubripes* (tru, FUGU 4.0), *Ciona intestinalis* (cin, KH), *Drosophila melanogaster* (dme, BDGP6), *Caenorhabditis elegans* (cel, WBcel235), and *Saccharomyces cerevisiae* (sce, R64-1-1).

We considered the downloaded data sets from Ensembl as reference, and did not account neither for intraspecies polymorphic variation nor for the quality of the genome assemblies.

### Glutamine Stretches and Codon Usage

We calculated the glutamine codon usage in all the retrieved data sets from Ensembl by counting the number of CAA and CAG triplets in pure Q stretches. The length of a Q stretch was taken as the number of consecutive glutamines, in a nonnested way (e.g., “QQQQ” was considered to be of length four glutamines, and not one time “QQQQ”, two times “QQ”, and four times “Q”).

The orthology information obtained from Ensembl was integrated to generate sets of orthologs per human protein. We took into account only the sets in which all nonhuman primates had at least one ortholog to the human protein. From them, we considered solely the sets in which at least one sequence had at least one region with four or more consecutive glutamines (supplementary file 1, Supplementary Material online). PolyQ regions from proteins with more than one glutamine stretch were analyzed independently. All the regions meeting this condition were manually verified, and were compared with the different aligned orthologous sequences.

To study the length of the Q stretches in the orthologs, we aligned them in UGENE v1.9.8 (Okonechnikov et al. 2012) using the T-Coffee algorithm with default parameters. To determine the length of a Q stretch, we counted the number of consecutive glutamines. An exception was made when two different Q stretches should have been considered in one sequence, and only one in an aligned orthologous region (e.g., “QQQQPQQQQ” in one protein aligned with “QQQQQQQQQ”). In that case, we counted the total number of glutamines in the aligned region, and not just the pure Q stretches (e.g., “QQQQPQQQQ” is considered to be of length eight glutamines and “QQQQQQQQQ” of nine glutamines); we did not analyze further the identity of the different amino acids present within the polyQ region. To study the glutamine codon usage in the orthologs, we followed the same procedure, counting the number of CAA and CAG triplets forming the Q stretches. In this case, we used the standalone version of TranslatorX (Abascal et al. 2010), with default parameters, to easily visualize the nucleotide alignments separated by codons.

The length of a Q stretch was considered to be stable if at least half of the orthologs had the same length; otherwise, it was considered unstable. In that case, we took as length of the unstable-in-length Q stretch the most frequent length among the orthologs. Given the case of two or more most frequent lengths, we took as the unstable length the most frequent one closest in evolution to human.

Reported *P* values are the result of a nonparametric Mann–Whitney *U* statistical test.

### Phylogenetic Relationships between Species

To assess the pairwise divergence time for each species and human, we obtained the estimated divergence time in million years given by the TimeTree database (Kumar et al. 2017). We used the phyloT tool version 2017.7 (http://phylot.biobyte.de/; last accessed February 26, 2018) to generate a phylogenetic tree to relate the organisms based on NCBI taxonomy.

### Proteins Related to polyQ-Associated Diseases

The amino acidic and nucleotidic sequences from the nine human proteins related to polyQ-associated diseases (Fan et al. 2014) (supplementary file 2, Supplementary Material online) were extracted from the downloaded data sets. Similarly, the orthologous sequences from the nonhuman primates were used. To complement the information about orthologs to those nine human proteins that were not defined by Ensembl, we conducted an additional procedure. We performed a BLAST search using the human protein as query versus the proteomes of the nonhuman primates with no defined ortholog (one search per human protein), using default parameters and low complexity filter off. As our only purpose here is to evaluate the length and codon usage of the one glutamine stretch associated to the disease, we considered a sequence as orthologous to the human query if their alignment covered the coordinates of the human disease-associated Q stretch. Fragments of orthologs not containing that Q stretch were thus not considered.

We followed the strategy explained above to evaluate the Q stretch length and codon usage of the full set of available orthologs to the nine human proteins related to polyQ-associated diseases.

## Results

### Glutamine Codon Usage Is Enriched in CAG Triplets in Longer Q Stretches

Amino acid codon usage has varied throughout evolution, and depend mostly on taxonomy. Here, we want to assess whether it is also influenced by the context of the surrounding codons. Focusing on the amino acid glutamine, Q, we calculated the frequency of glutamine stretches of different lengths in a set of 26 organisms representing major taxonomic groups, from the yeast *Saccharomyces cerevisiae* to the human proteome. For each stretch of consecutive glutamines in a proteome, we computed its length and the number of both CAA and CAG codons coding for the stretch (see Methods for details).

**Fig. 1.**
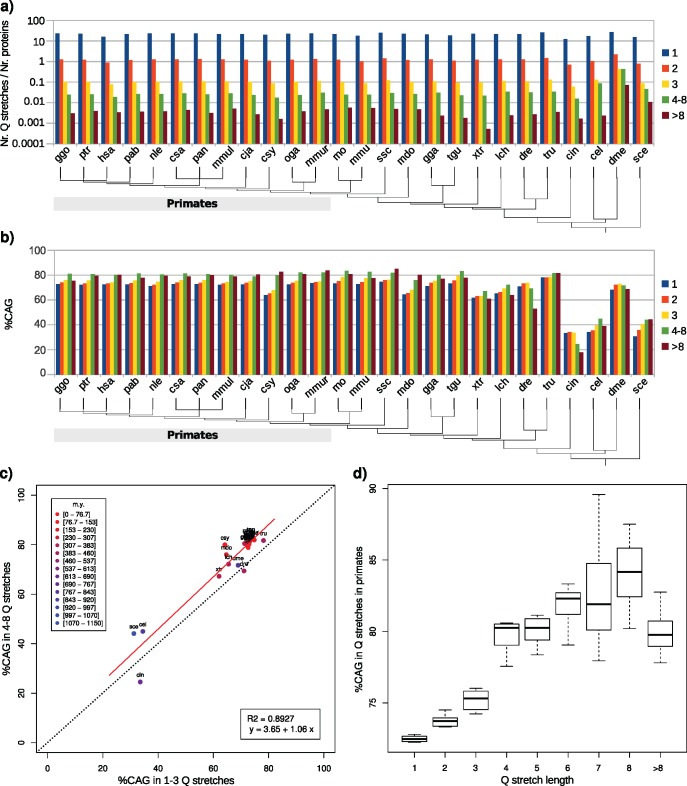
—Characterization of glutamine stretches in complete proteomes. (*a*) Number of glutamine stretches per number of proteins per proteome, depending on the stretch length. (*b*) Percentage of CAG triplets in glutamine stretches per proteome, depending on the stretch length. (*c*) Percentage of CAG triplets in glutamine stretches of lengths 4–8 compared with lengths 1–3; the result for each proteome is colored depending on the pairwise divergence time with human. The discontinuous line represents *x* = *y* values. (*d*) Overall CAG percentages in primates in glutamine stretches of varying lengths.

Glutamine stretches of short length are present in approximately similar numbers in all proteomes, and their proportion is extremely stable in primates (fig. 1*a*). There are in average more than ten “Q”, around one “QQ” and 0.1 “QQQ” per protein. It has been reported before that glutamines are coded by a ∼72% CAG in human (Athey et al. 2017); our results confirm it (fig. 1*b*, hsa), but solely in short glutamine stretches (1–3 Q). Glutamine stretches longer than three glutamines are coded by a higher CAG percentage. The larger proportion of 1–3 glutamine stretches (fig. 1*a*) bias the direct calculation of the glutamine codon usage. More distant-in-evolution species behave differently, with lower values of CAG percentages in 4–8 Q than in 1–3 Q in the zebrafish *Danio rerio* (dre) and the tunicate *Ciona intestinalis* (cin) (fig. 1*b*). The contrast of the percentage of CAG codons in 4–8 Q stretches versus in 1–3 Q stretches, that is, short polyQ and not polyQ, show a high correlation between these values when plotting the results for the 26 species (fig. 1*c*). Most of the species cluster in values of 70% CAG for 1–3 Q stretches and 80% CAG for 4–8 stretches, but *S. cerevisiae* (sce), *Caenorhabditis elegans* (cel), and *C. intestinalis* (cin). These three species are distant to human in evolution (676–1,105 Myr), which suggests the length-dependent glutamine codon usage was fixed after their speciation event.

Human and the rest of the nonhuman primates show similar proportions of glutamine stretches per protein, and also of the length-dependent CAG percentage, as described earlier. When itemizing the glutamine lengths from one to eight glutamines, and more than eight glutamines (fig. 1*d*), the triplet CAG is used in primates preferably in small polyQ (from a length of 1–3 to 4–8), whereas it is not abundant in grown homorepeats (>8 Q), for which positive selection for CAG codons might disappear. The trend in the CAG percentage values is clear, and show two well-defined groups of values (1–3 Q and >3 Q, *P* = 2.2E-16) consistent with our initial definition of what should be considered a polyQ.

### PolyQ Regions in Primates Are of Similar Length

No proteome-wide set of one-to-one orthologous sequences is available for a set of model organisms including human and other nonhuman primates. We built it by focusing on the set of primates studied in the previous section with information provided by Ensembl (see Methods for details); we used sets of orthologs in which all proteomes had at least one ortholog. A total of 8539 sets of orthologous sequences was initially obtained and then filtered to work only with those in which at least one protein from any of the organisms had at least one polyQ of length four or more, which resulted in 347 sets. From these, we identified 461 independent orthologous regions.

We counted the maximum number of consecutive glutamines in all the orthologous independent regions within the described data set, to account for both already-formed and for emerging/fading polyQ regions. This procedure allows us to characterize the length of a Q stretch in several points in evolution. As we are working with the full set of available completely sequenced primates, we are able to describe the evolutive drift of glutamine stretches in the last 74 million years in a comprehensive way.

**Fig. 2.**
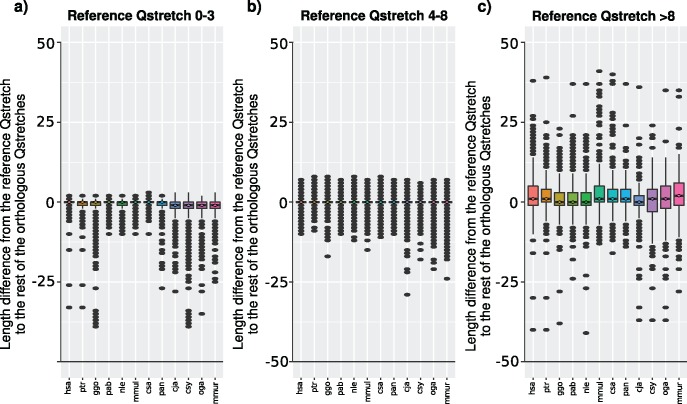
—Length differences of glutamine stretches between primates. Length differences from the reference glutamine stretch to the rest of the orthologous glutamine stretches, when the length of the reference stretch is (*a*) 0–3, (*b*) 4–8, and (*c*) >8.

For a given organism, we took all of its Q stretches as reference, and calculated the difference between their length and that of the rest of its orthologous regions. We repeated the procedure with the 12 primates, and split the results depending on the length of the reference Q stretch: 0–3 glutamines, 4–8 glutamines, and more than 8 glutamines (fig. 2). Both short Q stretches and polyQ regions (fig. 2*a* and *b*, respectively) show a general length similarity in all the species, with a very narrow length difference, especially in short polyQ. Short Q stretches are present in the results because at least one of its orthologous regions contain a polyQ, and thus logically they are generally either similar in length or shorter, meaning that either a few of the orthologous regions are a polyQ, or many of them, respectively. Short polyQ regions appear to be mostly stable-in-length. On the other hand, long polyQ regions are more unstable-in-length, although equally dissimilar in all organisms (fig. 2*c*). Glutamine homorepeats are then not significantly longer in human than in the rest of the nonhuman primates.

**Fig. 3.**
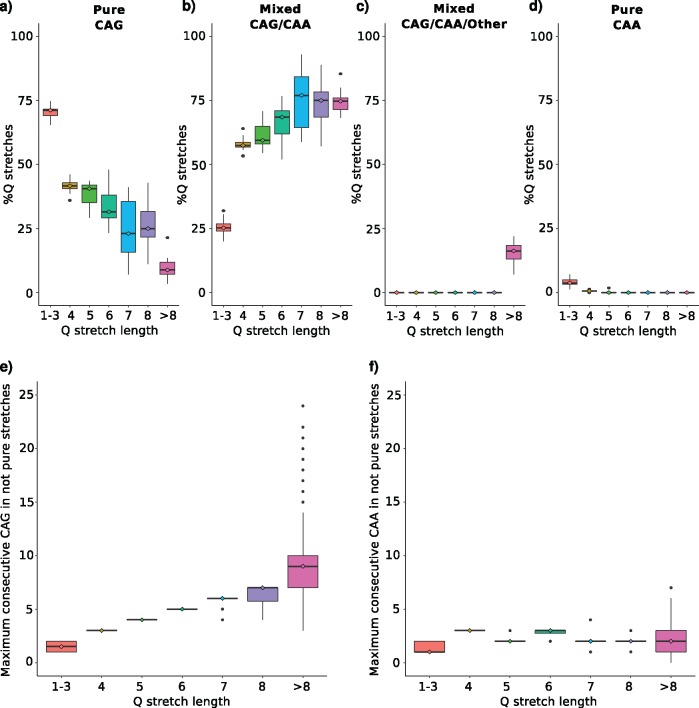
—Codon purity of glutamine stretches in primates. Percentage of glutamine stretches per length encoded by (*a*) only CAG codons, (*b*) a mix of CAG and CAA codons, (*c*) a mix of CAG, CAA, and other interrupting codons, and (*d*) only CAA codons. Considering only glutamine stretches encoded by more than one different triplet, maximum number of consecutive (*e*) CAG, and (*f*) CAA per length.

Most of the 461 orthologous polyQ regions are encoded by pure CAG codon stretches when short (fig. 3*a*) and mixed with CAA codons when long (fig. 3*b*). There are almost no CAA pure regions coding for a polyQ stretch (fig. 3*d*), and interruptions of different codons are also not frequent (fig. 3*c*). Finally, we calculated the longest run of consecutive CAG codons, and similarly of CAA codons, in stretches encoded by more than one different triplet. Consecutive CAA codon runs are always shorter than CAG consecutive stretches (fig. 3*e* and *f*). Both results hint at the use of CAA codons to disrupt long consecutive CAG stretches.

### Glutamine Codon Usage Is Enriched in CAG Triplets in Shorter Unstable-in-Length polyQ

The length stability of glutamine stretches was already briefly referred to in the previous section, by comparing the overall length differences in the full set of 461 independent orthologous regions. However, a one-by-one study of these regions is needed to assess their length-dependent stability and codon usage. We will not focus on polyQ growth or decrease, but in the stability of their length in the available data set. Both length growth and decrease would be influenced by the proteome taken as reference; however, the depiction of the length stability of glutamine stretches among primates is a property that takes into account all proteomes considered.

**Fig. 4.**
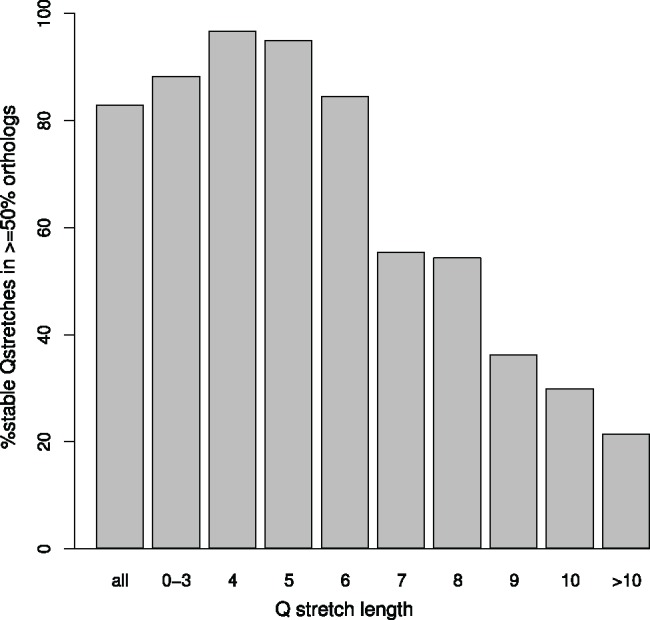
—Length-stability of glutamine stretches. Percentage of orthologous glutamine stretch regions with a stable length in at least half of the orthologs.

We considered a glutamine stretch to be stable-in-length if it had the same length in at least half of the orthologous regions. In that case, the stretch is labelled as stable and its length is taken as the one of the majority of them. Were the region unstable-in-length, its length would be taken as the most frequent among the orthologs (see Methods for details). As previously described (fig. 2), shorter Q stretches are more stable-in-length than longer ones (fig. 4). Stretches with more than ten glutamines (28/461 stretches) are rarely stable-in-length (21% of them). On the other hand, stretches of four consecutive glutamines (178/461 stretches) are almost always stable-in-length (97%). Results suggest that short polyQ appear to be generally held back within a controlled length range. They are most probably long enough to be functional, while not in risk of an unexpected expansion that could lead to instability and disease.

**Fig. 5.**
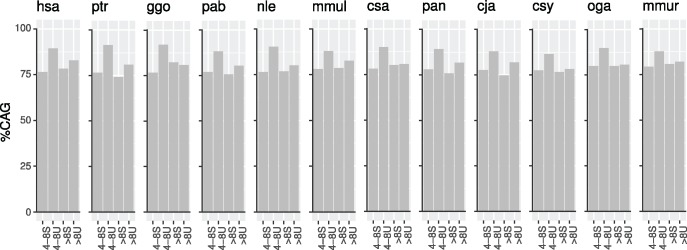
—Glutamine codon usage in stable- versus unstable-in-length polyQ. Codon usage calculated in stable-in-length (S) and unstable-in-length (U), short (4–8 Q), and long (>8 Q) polyQ stretches, in 12 primates.

Separate codon usage calculations in stable-in-length/unstable-in-length and short/long polyQ regions show that CAG is more frequent in short and unstable-in-length polyQ (“4-8U”) in all studied primates (fig. 5, *P* = 8.94E-08). This result suggests that CAG codons destabilize the glutamine stretch, probably assisting in the growth of the region.

### A Closer Look into the Proteins Related to polyQ-Associated Diseases

There are nine human proteins associated with diseases produced by the abnormal elongation of their polyQ regions. Following a similar procedure as the explained before, we checked both the polyQ length and glutamine codon usage in these proteins in 12 primates. The proteins in study are characterized for being pathological when surpassing an anomalous polyQ length threshold, specific for each protein. For example, the normal length of the glutamine repeat in human protein Huntingtin (EnsemblID: ENSP00000347184) is described to be 6-35, and in its pathological version 36-121 (Fan et al. 2014). It is important to notice that in this study we refer to the length of the polyQ region in the sequence obtained from the Ensembl database, which we take as reference; for Huntingtin, the sequence version present in Ensembl is 21 glutamines long.

All the proteins related to polyQ-associated diseases contain one polyQ region, but the androgen receptor (EnsemblID: ENSP00000363822), which contains three, with 23, 6, and 5 glutamines (in coordinates 58–80, 86–91, and 195–199, respectively). As the pathological stretch is the first of them, for the purpose of this work, we did not considered the second and the third regions.

Not every analyzed organism contains all of these nine proteins. The protein absences may be due to problems in the orthology mapping given by Ensembl, an erroneous genome sequencing or protein-coding gene annotation, or simply due to a gene loss event. We complemented the Ensembl orthology mapping with a manual strategy based on BLAST searches in the 12 proteomes to fill as much as possible the sets of orthologs for each protein (see Methods for details).

**Fig. 6.**
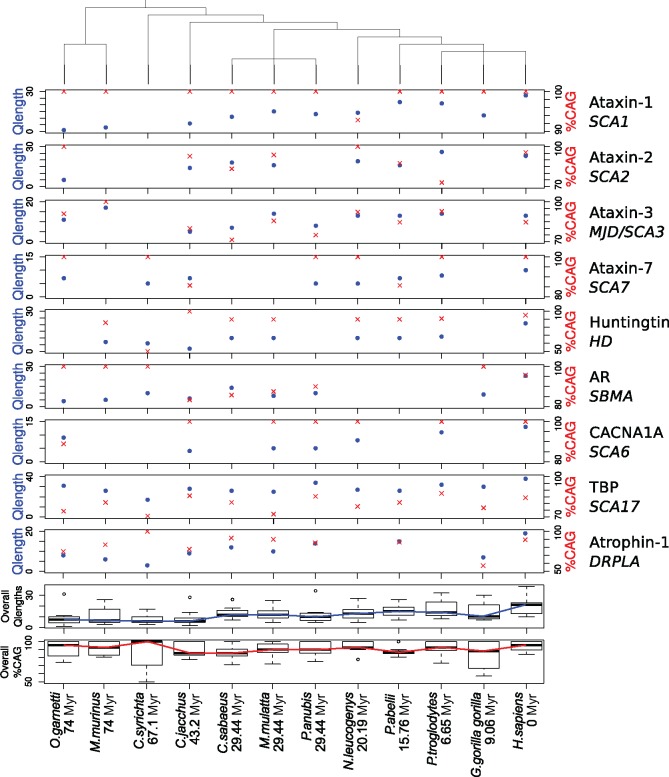
—PolyQ lengths and glutamine codon usage in proteins related to polyQ-expansion diseases. Divergence time for each organism and human is measured in million years (Myr). The tree on top relates the species based on their NCBI taxonomy. Each protein is appended with its related disease. The overall Q lengths (in blue) and CAG percentage prevalence (in red) plots take into account the results per species of the nine proteins shown above.

The overall length of the glutamine stretches in these proteins show that the human ones are generally longer (fig. 6, in blue); in fact, in seven out of nine sets of orthologous proteins, the human polyQ region is the longest one (not in Ataxin-2 and Ataxin-3). Nonhuman primates evolutionarily closer to human also have longer polyQ regions than more distant species. This is a deviation from the expected results: we have showed before that polyQ regions in primates are generally of similar length (fig. 2).

The overall proportion of CAG codons in those regions is unexpectedly high, with a mean value of >90% CAG in almost all species (fig. 6, in red). The CAG codon usage when computing all polyQ regions with more than eight glutamines was calculated to be between 75% and 80% for all primates (fig. 5). The extreme CAG codon presence in the polyQ regions of proteins related to polyQ-associated diseases may produce an instability that boosts the CAG-dependent polyQ growth in evolution, explaining the growth pattern of the polyQ regions in these proteins from evolutionarily distant nonhuman primates to human (supplementary fig. 1, Supplementary Material online, *P* value = 0.007). The differences at the level of nucleotides (higher CAG triplet proportion than the background) and amino acids (longer glutamine stretches in human and evolutionarily closer nonhuman primates) may explain the association of these nine proteins with human diseases.

## Discussion

This work presents a comprehensive evolutionary characterization of homoglutamine repeats in both amino acidic and nucleotidic contexts. We have showed that for all studied species the glutamine codon usage depends on the number of consecutive glutamines in a stretch, being in most of the species enriched in CAG triplets in longer Q stretches (fig. 1). Primates present a direct correlation between the number of consecutive glutamines in a stretch and the percentage of CAG triplets coding them, covering glutamine stretches with lengths 1–3 and short polyQ with lengths 4–8. Once the polyQ region is established and long enough, the presence of CAG is not required anymore. Our results suggest the greater importance of CAG triplets in generating the polyQ region than in elongating it once it reaches a certain length threshold. This result is supported by the fact that orthologous short unstable-in-length polyQ regions in primates are enriched in CAG (fig. 5, data labels “4-8U”).

Orthologous glutamine stretches in primates are generally of a similar length (fig. 2). The length range of orthologous regions to stretches of length 4–8 is very narrow, which is confirmed by the greater length stability of shorter glutamine stretches (fig. 4). In the same way, longer glutamine stretches are more unstable-in-length, but they are not significantly longer in any species. Contravening this result, polyQ regions of proteins related to polyQ-associated diseases are unexpectedly longer in human and evolutionarily closer nonhuman primates (fig. 6). They also deviate from the proteome-wide codon usage of glutamine stretches, showing an overall higher CAG proportion in almost all species.

CAA codons serve as disruptors of long pure CAG stretches (fig. 3), which may be selected for to avoid the uncontrollable growth of these regions produced by CAG expansion through slippage-related mechanisms (Kraus-Perrotta and Lagalwar 2016; Ciesiolka et al. 2017). The smaller amount of CAA triplets encoding for polyQ regions associated to polyQ diseases suggests a role for CAA codons as phenotype modulators. Even though the frequency of codons different to CAA interrupting consecutive CAG stretches is low, is has been previously reported a role of these interruptions evading homologous DNA recombination (Barik 2017), slowing the aggregation rates of polyQ regions, decreasing fiber formation rates, increasing oligomer stability (Menon et al. 2013), and preventing CAG expansion (Ciesiolka et al. 2017). Whether the phenotypic outcome of an interruption in a long CAG stretch produced by a CAA (silent mutation) or by another codon (missense mutation) is different remains to be deciphered.

Our interest in the proteins related to polyQ-associated diseases is anthropocentric, as they are associated with neurological diseases described in human; there are probably more proteins in nonhuman primates associated with neurodegenerative diseases in them which we do not know of because of their nonpathogenicity in human.

To suggest an evolutionary model to explain the glutamine codon usage in polyQ regions in primates, we point to the following observations. First, the percentage of CAG triplets coding for glutamine stretches depends on the number of consecutive glutamines (fig. 1*d*): lower percentages for 1–3 Q, higher percentages for 4–8 Q, and medium percentages for >8 Q. Second, polyQ length is generally stable across orthologs (fig. 2). Third, shorter polyQ are more stable-in-length than longer ones (fig. 4). Fourth, CAG codons are associated with length instability (fig. 5, “4-8S” vs “4-8U”). Fifth, much higher percentages of CAG than expected for their length are present in polyQ regions of proteins related to polyQ-associated diseases (fig. 6 “Overall %CAG” vs fig. 1*d* “>8”), and they present an overall polyQ length longer in human and evolutionary-related nonhuman primates and shorter in species more distant in evolution (fig. 6). We propose then that the observations summarized above collectively suggest the following evolutionary model for polyQ in primates: 1) CAG are positively selected in evolution to generate a short polyQ region from a short glutamine stretch; 2) a short polyQ region is probably long enough to be functional, and thus their growth is no longer selected; 3) as a mechanism to stop longer polyQ to keep growing and to reduce instability, CAG triplets are either actively counter selected or in neutral evolution; 4) longer polyQ regions escaping this blockage may grow uncontrollably and be involved in the development of polyQ-associated diseases.

The validity of the presented model needs to be tested in vivo. Even if the model is thought to explain the glutamine codon usage in polyQ regions in primates, a more-distant species with a shorter lifespan could be used to test our hypothesis. For example, by integrating in its genome polyQ tracts with various CAG percentages, and checking in successive generations if the glutamine stretches grow in a CAG-dependent way. The yeast *S. cerevisiae* has already been used to express fragments of Huntingtin with polyQ expansions to study polyglutamine toxicity (Krobitsch and Lindquist 2000; Duennwald et al. 2006), therefore we propose it as a potential model organism to prove our model. Furthermore, future gene therapies may induce point mutations in polyQ regions to transform CAG codons into CAAs, which could stop abnormal CAG-mediated expansions of glutamine tracts.

With this work, we hope to raise awareness to the usefulness of studying homorepeat evolution. Gathering information from a data set of complete proteomes, we could show that, while in primates polyglutamines are rather stable-in-length, they evolve in a CAG-dependent manner. Further efforts should be made to research the evolution of other homorepeats, taking advantage of the growing collection of complete proteomes available in public databases.

## Supplementary Material

Supplementary data are available at *Genome Biology and Evolution* online.

## Supplementary Material

Supplementary Data
