# Supplementary material for: Glutamine Codon Usage and polyQ Evolution in Primates Depend on the Q Stretch Length
Source: Genome Biol Evol. 2018 Mar 1;10(3):816–25. doi: 10.1093/gbe/evy046 (PMC5841385; doi:10.1093/gbe/evy046)
Supplement: Supplementary Data [file evy046_supp.pdf]

Supp.Fig1. Proteome-wide characterization of %CAG codon usage per polyQ length, in twelve primates (white). Showcased are the values for homorepeats related to polyQ-expansion diseases from human (in green) and nonhuman primates (in red).

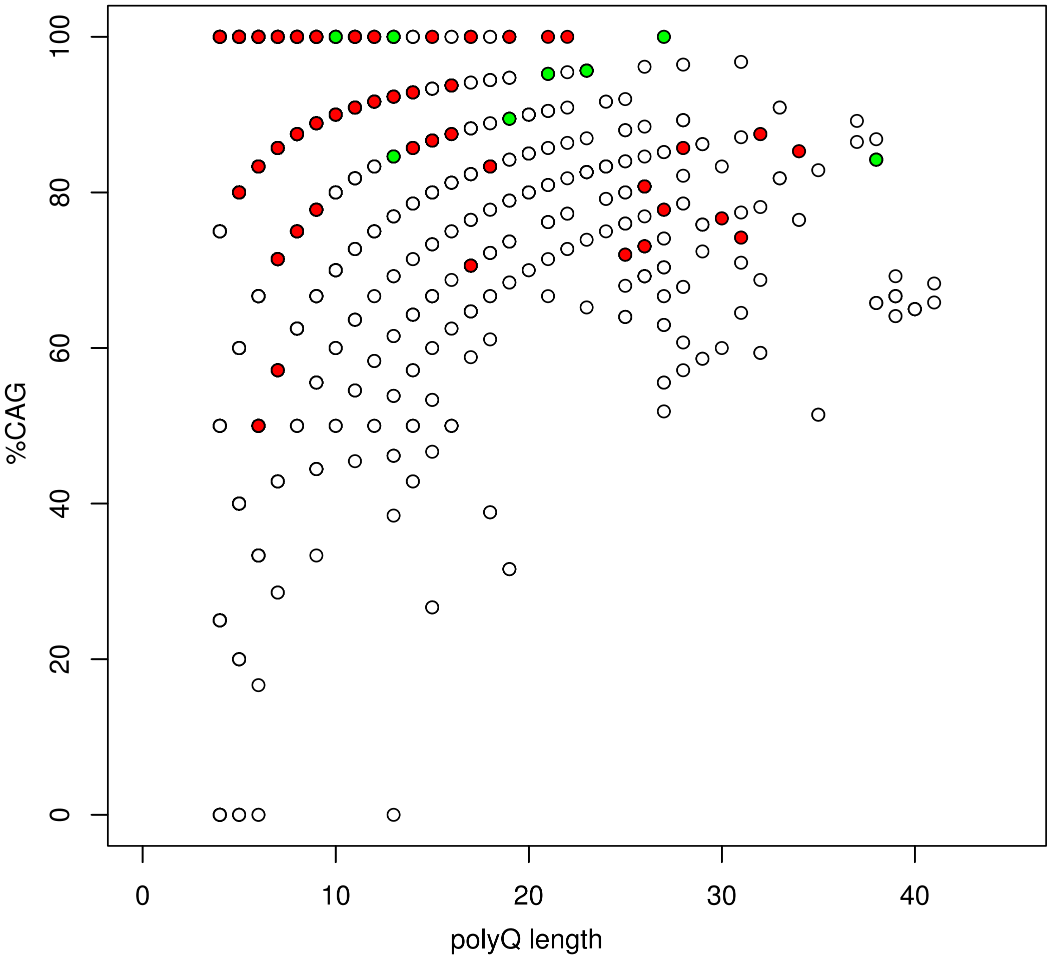

Supp.File1. Set of human proteins for which itself or at least one of its orthologs has at least one region with four or more consecutive glutamines.

ENSP00000011619
ENSP00000171887
ENSP00000176763
ENSP00000202677
ENSP00000204517
ENSP00000205061
ENSP00000215793
ENSP00000216034
ENSP00000217086
ENSP00000219746
ENSP00000219905
ENSP00000223145
ENSP00000225388
ENSP00000230658
ENSP00000231524
ENSP00000240874
ENSP00000242057
ENSP00000244769
ENSP00000245564
ENSP00000251020
ENSP00000251038
ENSP00000251636
ENSP00000252891
ENSP00000253031
ENSP00000254616
ENSP00000256495
ENSP00000257118
ENSP00000257915
ENSP00000258399
ENSP00000260210
ENSP00000261523
ENSP00000262041
ENSP00000262189
ENSP00000262367
ENSP00000263062
ENSP00000263208
ENSP00000263253
ENSP00000264606
ENSP00000264670
ENSP00000264731
ENSP00000264883
ENSP00000264956
ENSP00000265069
ENSP00000265271
ENSP00000265433
ENSP00000268124
ENSP00000268711
ENSP00000268712
ENSP00000269844
ENSP00000269980
ENSP00000273317
ENSP00000273668
ENSP00000274853
ENSP00000276079
ENSP00000276420
ENSP00000277225
ENSP00000277541
ENSP00000280665
ENSP00000281708
ENSP00000283147
ENSP00000284320
ENSP00000285199
ENSP00000285871
ENSP00000286835

ENSP00000290607
ENSP00000295550
ENSP00000297338
ENSP00000300231
ENSP00000301067
ENSP00000305255
ENSP00000305976
ENSP00000307183
ENSP00000307479
ENSP00000308533
ENSP00000308741
ENSP00000309555
ENSP00000310301
ENSP00000313199
ENSP00000313490
ENSP00000314343
ENSP00000320485
ENSP00000320503
ENSP00000321826
ENSP00000323065
ENSP00000324463
ENSP00000327025
ENSP00000327077
ENSP00000328511
ENSP00000329357
ENSP00000330753
ENSP00000332706
ENSP00000334319
ENSP00000334474
ENSP00000335044
ENSP00000335500
ENSP00000338173
ENSP00000338185
ENSP00000339933
ENSP00000340507
ENSP00000341282
ENSP00000341292
ENSP00000341957
ENSP00000342755
ENSP00000343377
ENSP00000343535
ENSP00000343819
ENSP00000344219
ENSP00000344546
ENSP00000345216
ENSP00000345702
ENSP00000346359
ENSP00000347046
ENSP00000347184
ENSP00000347823
ENSP00000348416
ENSP00000349016
ENSP00000349748
ENSP00000350719
ENSP00000351141
ENSP00000351407
ENSP00000351416
ENSP00000351686
ENSP00000352314
ENSP00000352463
ENSP00000354501
ENSP00000354669
ENSP00000354896
ENSP00000355133
ENSP00000355925
ENSP00000355961
ENSP00000356172

ENSP00000357129
ENSP00000357440
ENSP00000357474
ENSP00000357679
ENSP00000357880
ENSP00000357907
ENSP00000358064
ENSP00000358813
ENSP00000359042
ENSP00000359474
ENSP00000360493
ENSP00000360899
ENSP00000361027
ENSP00000361066
ENSP00000361209
ENSP00000361278
ENSP00000361467
ENSP00000361824
ENSP00000362238
ENSP00000362441
ENSP00000362814
ENSP00000363822
ENSP00000363929
ENSP00000364324
ENSP00000364449
ENSP00000364815
ENSP00000365007
ENSP00000365188
ENSP00000365318
ENSP00000366070
ENSP00000366843
ENSP00000367038
ENSP00000367343
ENSP00000367923
ENSP00000369473
ENSP00000369756
ENSP00000369887
ENSP00000370695
ENSP00000370938
ENSP00000371155
ENSP00000374213
ENSP00000374529
ENSP00000375942
ENSP00000376154
ENSP00000377721
ENSP00000378431
ENSP00000378721
ENSP00000378830
ENSP00000379144
ENSP00000379396
ENSP00000379401
ENSP00000379644
ENSP00000380019
ENSP00000380136
ENSP00000380888
ENSP00000381007
ENSP00000382382
ENSP00000382767
ENSP00000384004
ENSP00000384048
ENSP00000384109
ENSP00000384160
ENSP00000384484
ENSP00000384792
ENSP00000384823
ENSP00000385450
ENSP00000385571

ENSP00000386049
ENSP00000386200
ENSP00000386456
ENSP00000386759
ENSP00000387020
ENSP00000387170
ENSP00000391504
ENSP00000391723
ENSP00000392028
ENSP00000398824
ENSP00000399518
ENSP00000399968
ENSP00000400921
ENSP00000401678
ENSP00000402935
ENSP00000404049
ENSP00000408617
ENSP00000413418
ENSP00000414516
ENSP00000417003
ENSP00000417235
ENSP00000417510
ENSP00000417583
ENSP00000419361
ENSP00000419465
ENSP00000420095
ENSP00000420194
ENSP00000420294
ENSP00000420875
ENSP00000421180
ENSP00000425133
ENSP00000427018
ENSP00000430497
ENSP00000433415
ENSP00000434359
ENSP00000439585
ENSP00000439689
ENSP00000440207
ENSP00000440674
ENSP00000441823
ENSP00000443176
ENSP00000443246
ENSP00000443985
ENSP00000447488
ENSP00000448073
ENSP00000449396
ENSP00000452454
ENSP00000453095
ENSP00000459626
ENSP00000464443
ENSP00000475384
ENSP00000477878
ENSP00000478320
ENSP00000479510
ENSP00000482128
ENSP00000482229
ENSP00000483254
ENSP00000483667
ENSP00000484803
ENSP00000486610
ENSP00000489829
ENSP00000490530
ENSP00000490726
ENSP00000491215
ENSP00000491841
ENSP00000307093
ENSP00000383920

ENSP00000462945
ENSP00000469534
ENSP00000258301
ENSP00000377298
ENSP00000378307
ENSP00000398610
ENSP00000427120
ENSP00000295851
ENSP00000307508
ENSP00000437464
ENSP00000287394
ENSP00000297788
ENSP00000315371
ENSP00000373347
ENSP00000217026
ENSP00000265381
ENSP00000330601
ENSP00000339587
ENSP00000350425
ENSP00000370736
ENSP00000410257
ENSP00000428564
ENSP00000434466
ENSP00000405405
ENSP00000352572
ENSP00000258243
ENSP00000262519
ENSP00000283296
ENSP00000307411
ENSP00000329995
ENSP00000331268
ENSP00000340445
ENSP00000350036
ENSP00000351113
ENSP00000395772
ENSP00000482027
ENSP00000199448
ENSP00000223023
ENSP00000234739
ENSP00000262507
ENSP00000276893
ENSP00000313420
ENSP00000318982
ENSP00000343658
ENSP00000375863
ENSP00000408295
ENSP00000435365
ENSP00000470082
ENSP00000476519
ENSP00000253048
ENSP00000266070
ENSP00000273963
ENSP00000275517
ENSP00000296043
ENSP00000296137
ENSP00000313140
ENSP00000360797
ENSP00000377797
ENSP00000378917
ENSP00000379156
ENSP00000385215
ENSP00000387361
ENSP00000387362
ENSP00000395723
ENSP00000401371
ENSP00000402515
ENSP00000436773

ENSP00000235628
ENSP00000257570
ENSP00000261778
ENSP00000270517
ENSP00000288710
ENSP00000329360
ENSP00000341030
ENSP00000353098
ENSP00000353670
ENSP00000363944
ENSP00000366757
ENSP00000368022
ENSP00000371471
ENSP00000412724
ENSP00000485238

Supp.File2. Set of human proteins related to polyQ-associated diseases.

ENSP00000244769
ENSP00000366843
ENSP00000339110
ENSP00000295900
ENSP00000353362
ENSP00000230354
ENSP00000347184
ENSP00000349076
ENSP00000363822
